# Complete Genome Sequence of Thermophilic Bacterium Aeribacillus pallidus PI8

**DOI:** 10.1128/MRA.00224-20

**Published:** 2020-04-23

**Authors:** Kyosuke Kita, Atsushi Ishida, Kosei Tanaka, Shu Ishikawa, Ken-ichi Yoshida

**Affiliations:** a Department of Science, Technology and Innovation, Kobe University, Kobe, Hyogo, Japan; University of Southern California

## Abstract

Here, we report the complete genome sequence of Aeribacillus pallidus PI8, a thermophilic bacterium, isolated from soybean stem extract. The sequence was determined using Illumina and Nanopore sequencers. Bioinformatic analyses of the genome sequence revealed the presence of possible bacteriocin gene clusters.

## ANNOUNCEMENT

Aeribacillus pallidus is a Gram-positive, aerobic, and endospore-forming thermophilic bacterium previously reclassified from the genus *Geobacillus* (phylum *Firmicutes*, family *Bacillaceae*) (1). We were interested in an unknown thermophilic bacterium found in a hot water extract of soybean stems harvested from Nakasatsunai, Hokkaido, Japan, and characterized it as a novel strain of *A. pallidus* as follows.

A total of 400 g of a fresh soybean stem was milled and boiled in 400 ml of water for 1 h, which was subjected to a diatomaceous earth filtration to prepare the extract. To purify pinitol (3-*O*-methyl-d*-chiro*-inositol) from the extract (in order to degrade all the sugars except pinitol), we inoculated the non-inositol-degrading strain PS8 of Geobacillus kaustophilus (2) into the extract and allowed it to grow at 55°C overnight with shaking at 180 rpm. The extract had not been autoclaved, which might have permitted some contaminant species. An aliquot of the culture was spread onto LB plates (3) and incubated at 55°C overnight to result in at least two types of bacterial colonies, including white ones of an unknown species and brown ones of PS8. In order to characterize the unknown species forming the white colonies, one white colony was isolated and inoculated into LB liquid medium to grow at 55°C overnight with shaking at 180 rpm. The cells in the liquid culture were harvested, and their genomic DNA (gDNA) was prepared via conventional phenol-chloroform extraction (4) to amplify and sequence its 16S rRNA genes. The species was classified as Aeribacillus pallidus, as described previously (1), and designated strain PI8.

Next, a gDNA library of PI8 was prepared for Illumina sequencing using the NEBNext Ultra DNA library prep kit for Illumina (New England BioLabs). Sequencing was carried out on a MiSeq platform using the MiSeq reagent kit v3 (2 × 300-bp paired ends). For Nanopore sequencing, another gDNA library was prepared using a rapid barcoding kit (catalog number SQK-RBK004) and sequenced on the MinION instrument (R9.4 SpotON FLO-MIN106 flow cell). The reads were base called by Albacore v.2.2.8 and demultiplexed and trimmed using Porechop v.0.2.4 with the default settings (https://github.com/rrwick/Porechop).

Overall, 2 × 3,845,275 Illumina sequencing reads and 123,381 Nanopore sequencing reads were assembled by Unicycler v.0.4.7 (5) without Pilon polishing. The assembled sequences were further refined by unicycler_polish (Unicycler package v.0.4.7) using Illumina short reads. Final analysis of the whole genome sequence indicated that PI8 is a novel isolate with a single 3,833,114-bp-long circularized chromosome containing a G+C content of 39.0%. The final coverage of the genome was 542×. Annotation was performed using DFAST v.1.1.0 (6) and revealed 3,718 coding sequences, 20 rRNA sequences, and 84 tRNA sequences. Default parameters were used for all software unless otherwise specified as above.

Bacteriocins are antimicrobial peptides produced by various bacteria, including strains of *A. pallidus* (7). The PI8 genome sequence was submitted to the BAGEL4 Web server (8) to search for bacteriocin gene clusters with the default settings. The search identified three genetic loci, namely, (1) a locus related to the biosynthesis of a sactipeptide (coding DNA sequence [CDS], APP_26820), (2) a locus coding a sporulation killing factor (APP_02670), and (3) a locus coding an amylocyclicin-like circular bacteriocin (APP_08000) with 45.3% identity to amylocyclicin (9), based on BLAST alignment (10). In the future, we will identify which of these loci are involved in its possible production of antibacterials.

### Data availability.

The complete genome sequence of *A. pallidus* PI8 has been deposited in DDBJ/ENA/GenBank under the accession number AP022323. The raw sequence data are available under SRA accession numbers DRX202495 and DRX202750.
